# Treating Diabetes Utilizing a Low Carbohydrate Ketogenic Diet and Intermittent Fasting Without Significant Weight Loss: A Case Report

**DOI:** 10.3389/fnut.2021.687081

**Published:** 2021-06-28

**Authors:** Kristen Gavidia, Tro Kalayjian

**Affiliations:** ^1^Touro College of Osteopathic Medicine, New York, NY, United States; ^2^Yale New Haven Health System, New Haven, CT, United States

**Keywords:** diabetes, ketogenic, fasting, nutrition, A1C, insulin resistance, low carbohydrate

## Abstract

Prediabetes and diabetes are leading causes of morbidity and mortality in the United States and are growing in prevalence up to 45% of the population over the past 50 years. Current guidelines from the ADA recommend focusing on energy balance, portion sizes, and weight loss while cautioning that no ideal macronutrient composition has been determined. The guidelines also do not recommend intermittent fasting. In contrast, we report three cases of a substantial reduction in A1C without clinically significant weight loss using a unique, patient-centered program that utilizes low carbohydrate diets with intermittent fasting. These results call into question the role of weight reduction in the management of diabetes while highlighting the unique importance of carbohydrate restriction and intermittent fasting. In this study, we demonstrate a case series of three patients with a substantial reduction in A1C and significantly reducing the need for pharmacotherapy without clinically significant weight loss. Although anecdotal, these results call into question the emphasis of ADA on weight reduction and energy intake reduction for the management of diabetes.

## Introduction

Prediabetes and diabetes are medical conditions characterized by high glucose levels and insulin resistance afflicting 45% of the population in the United States (1). Modern, ultra-processed food and the resulting obesity epidemic have been largely attributed to this growing prevalence. Longstanding complications that arise from this disease include retinopathy, peripheral neuropathy, and diabetic nephropathy (2). Many patients rely on diabetic medications to control their glucose levels and to reduce their hemoglobin A1C level, which is a 3-month average of blood glucose. To reduce the burden placed on compliance with medication, the American Diabetes Association (ADA) released guidelines for nutrition therapy of diabetes management which emphasizes on weight loss and reduction in energy intake while recommending against a focus on dietary macronutrient composition or meal timing (3). The definition of ADA of clinically significant weight loss is at least a 5% reduction in weight in patients with concurrent obesity and is associated with producing beneficial outcomes in glycemic control and lipids (4). The ADA also highlights that more intensive weight loss goals upward of 15% may be appropriate to maximize benefit.

In this case series, we present three patients with diabetes who were all on medication management including insulin and metformin at the beginning of the program. The program took place in an outpatient clinic and was monitored and implemented by a physician and two health coaches. Before beginning the weight loss program, each patient was screened for anorexia and bulimia. Each patient was educated on various eating patterns, of which each patient self-selected a low-carbohydrate diet. Patients were also provided education regarding time-restricted eating, various fasting regimens, and risks and benefits were discussed. At all points, informed consent was provided and the autonomy of patients was respected. A low carbohydrate, the ketogenic diet was initially implemented for all three patients and the daily requirement of carbohydrates were generally kept under 30 g. After 6 weeks, a daily fasting pattern was recommended to patients. They were instructed to consider from 18 to 23 h of daily intermittent fasting, at their discretion, if they subjectively felt that their hunger and appetites were suppressed. The counseling provided to the patient was to track carbohydrate counts, self-perception of appetite and hunger, and calorie counting was specifically discouraged.

The patients were provided with a FreeStyle Libre continuous glucose monitor (CGM), which is placed on their upper arm at the clinic. Real-time continuous glucose data were available to both patients and remotely to the clinic staff. A QardioBase bioimpedance scale and QardioArm blood pressure cuff were also provided to each patient to remotely track their daily weight, body composition data, and blood pressure. Laboratory tests were performed every 2 months which included hemoglobin A1C, lipid panel, serum or breath ketones, complete metabolic profile, and complete blood count. A combination of both in-office and virtual visits was conducted to monitor the progress of each patient. During this program, no adverse events were reported. The focus of this case series is to present the weight and glycemia results of three patients.

## Results

At the end of the 4 months, the average levels of A1C dropped 5.2 points from 11.9 to 6.7, and the average weight loss was 8.7 pounds in all three patients while discontinuing all diabetic medications, including insulin and metformin. In these patients, consistent with the literature, we noted favorable changes in high-density lipoprotein cholesterol and triglycerides values and unfavorable changes in calculated low-density lipoprotein cholesterol (5). These findings were closely monitored and explained to patients for shared decision-making. All of the patients were producing ketones.

### Patient #1

**Figure 1:**
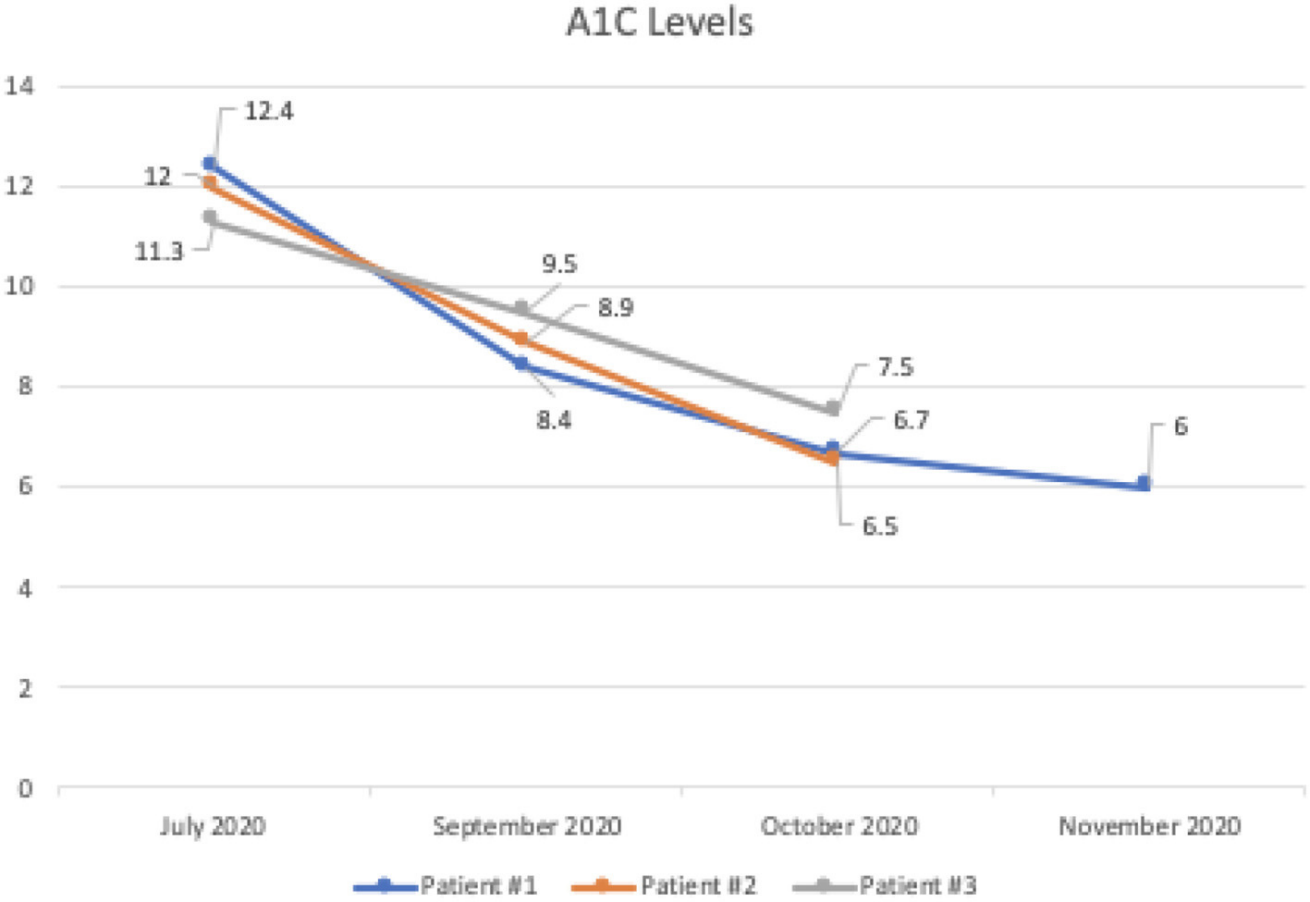
A1C levels of all three patients during the program.

**Figure 2:**
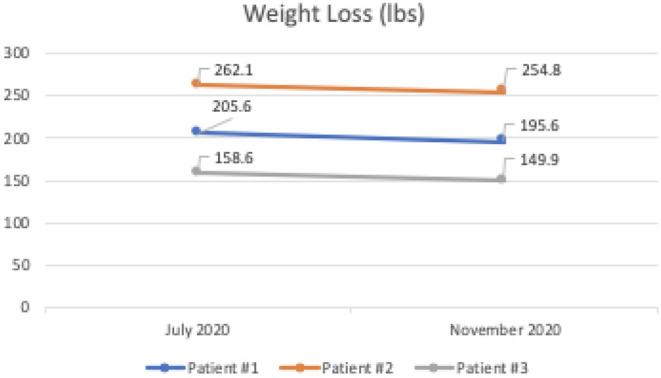
Weight loss (lbs) of all three patients during the program.

**Figure 3:**
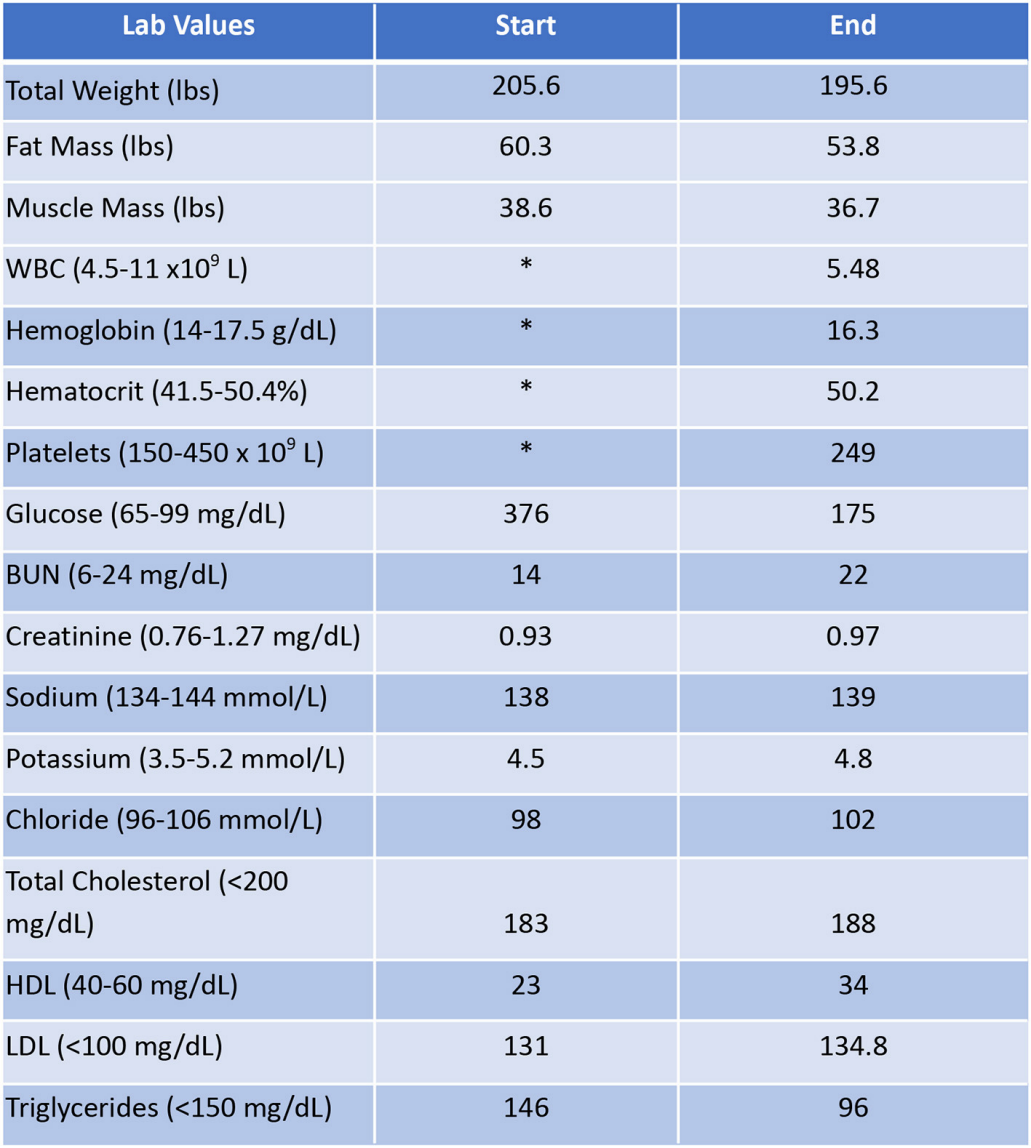
Lab values of Patient #1 at the start and the end of the program. ^*^Sample was hemolyzed.

**Figure 4:**
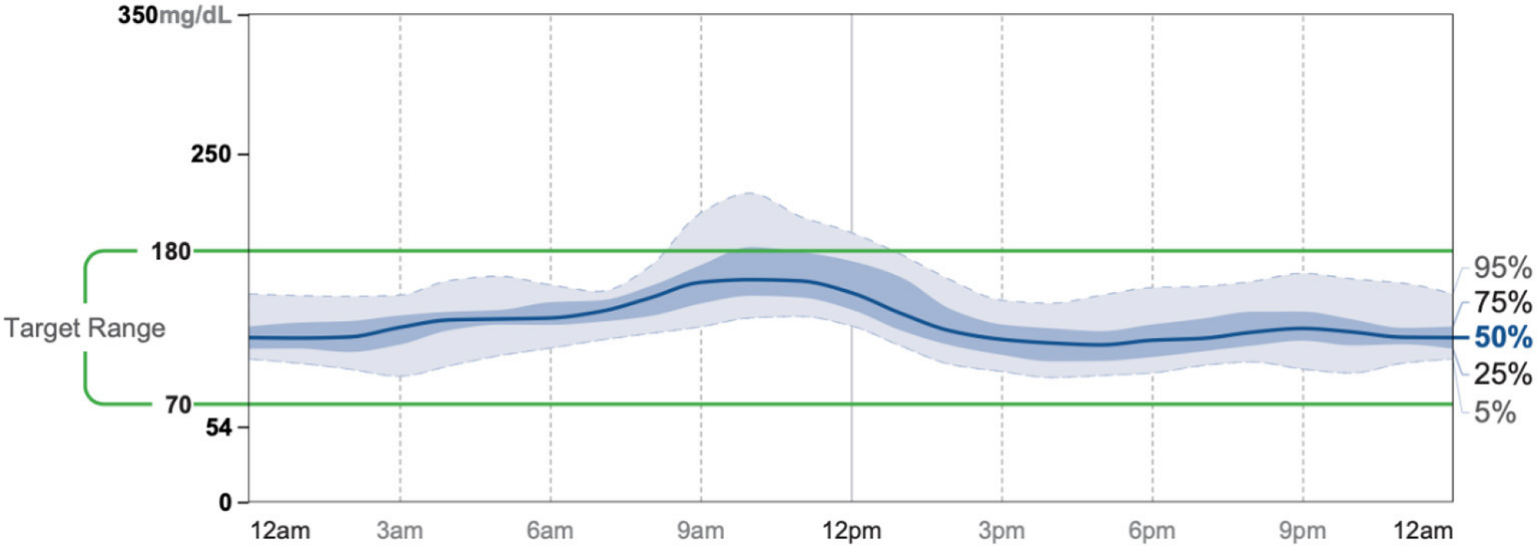
AGP 7/28-8/10.

**Figure 5:**
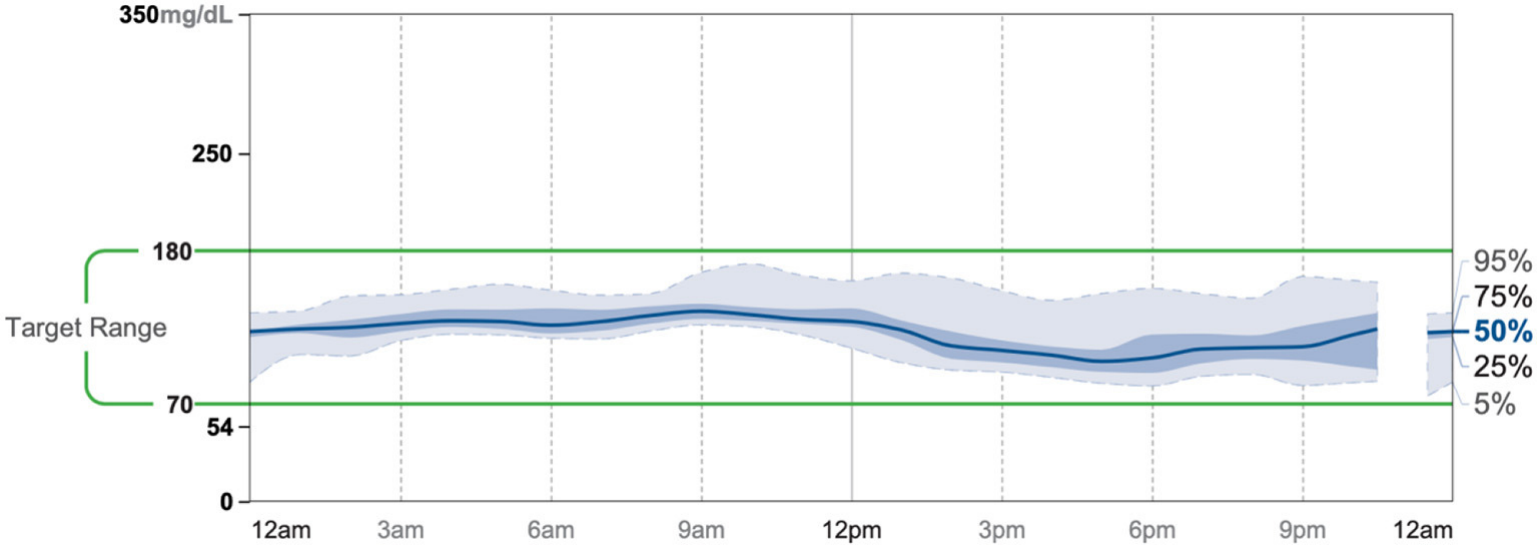
AGP 11/25-12/8.

Patient #1 is a 63-year-old Caucasian male with a history of diabetes from the maternal family. He was taking insulin and metformin of 1,000 mg to manage his condition. After the first appointment, he was taken off insulin and had his metformin lowered to 750 mg. The importance of family support was emphasized during visits, and he chose a low carbohydrate, ketogenic diet along with IF. In about 4 months, he was taken off from metformin, had his A1C drop significantly from 12.4 to 6 as shown in Figure 1, and lost 10 pounds from 205.6 to 195.6 pounds as shown in Figure 2. Figure 3 shows the lab values of the patient from start to end of the program. Of note, his glucose improved from 376 to 175, HDL improved from 23 to 34, and triglycerides decreased from 146 to 96. The patient had capillary ketones checked in the office and were present throughout the program. During this program, his AGP report showed an improvement of his average glucose levels in Figures 4, 5.

### Patient #2

**Figure 6:**
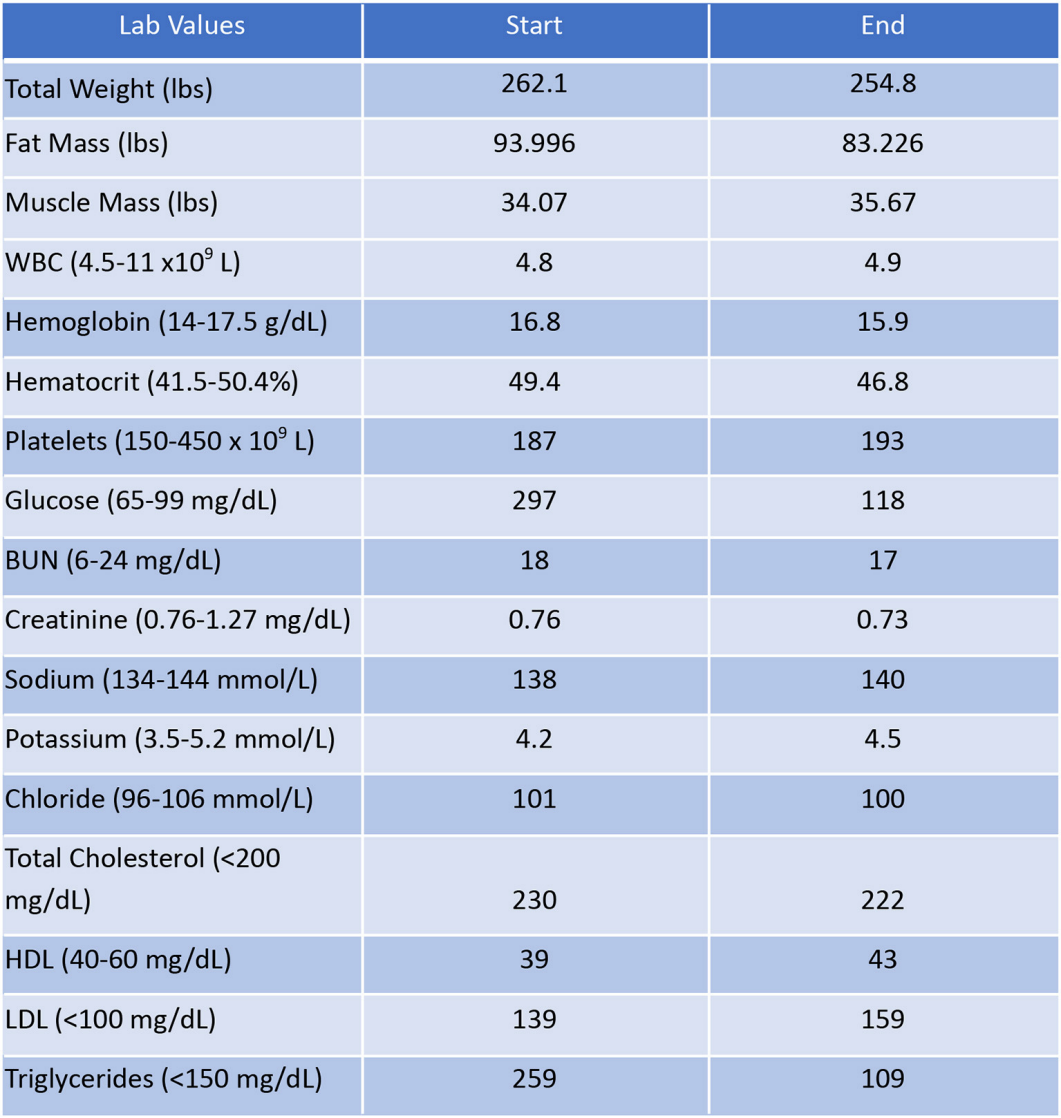
Lab values of Patient #2 at the start and the end of the program.

**Figure 7:**
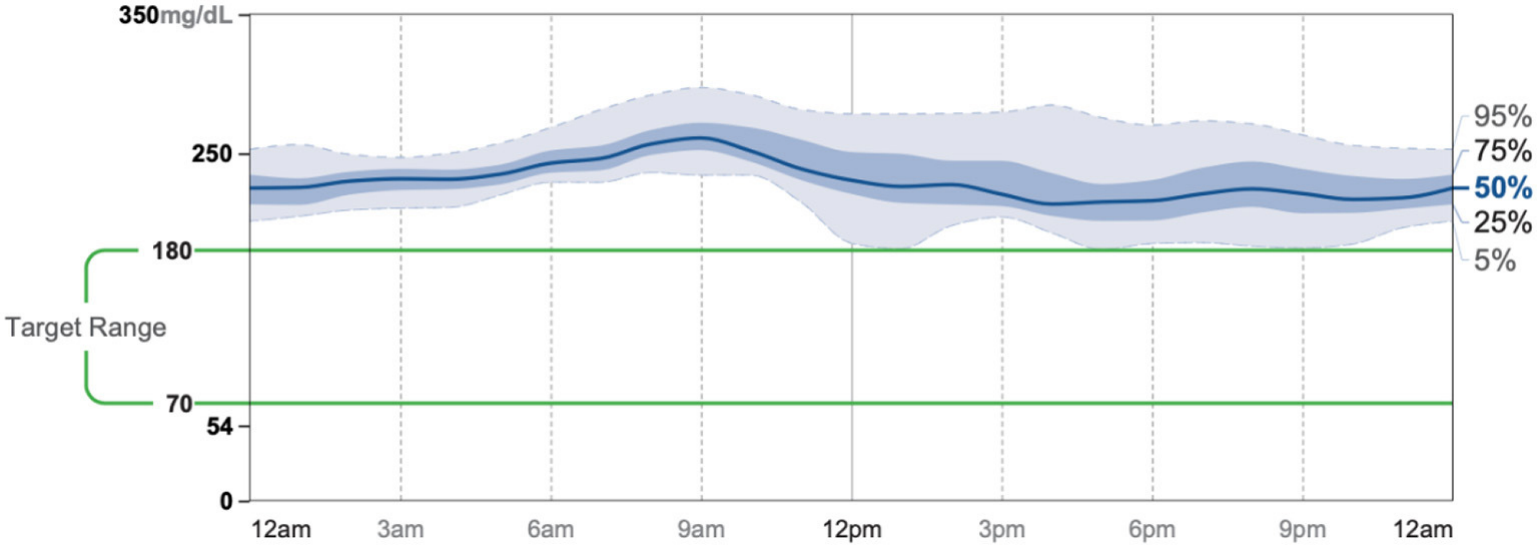
AGP 7/19-8/1.

**Figure 8:**
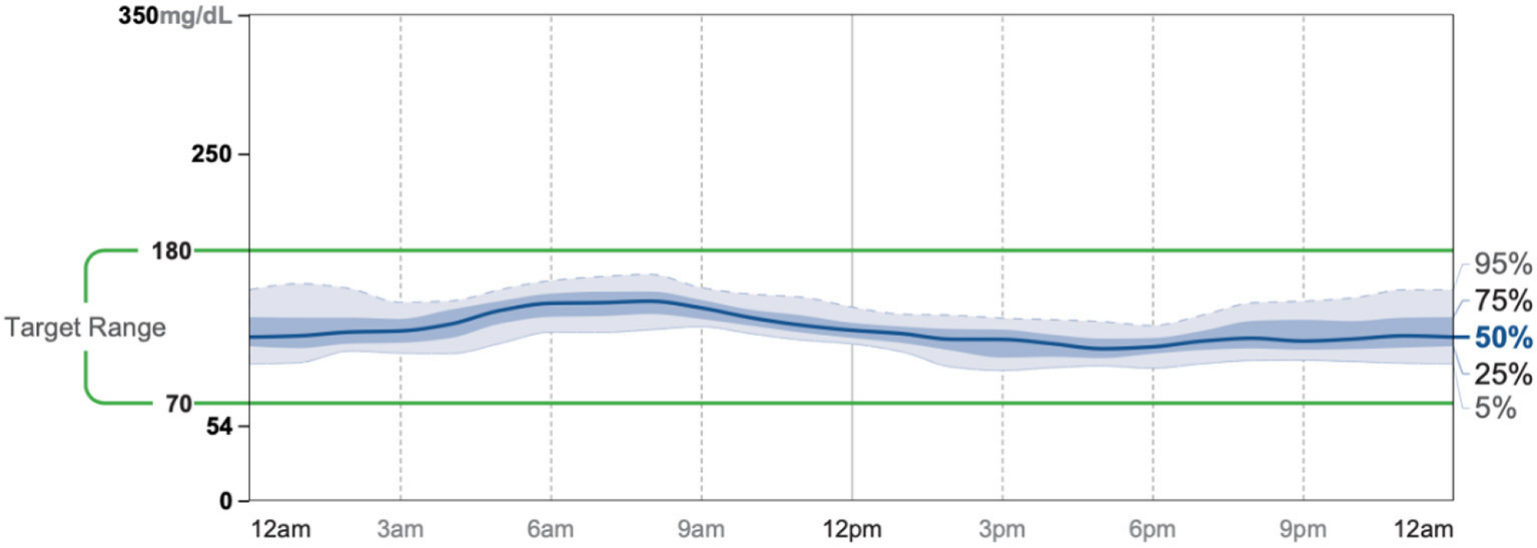
AGP 11/5-11/18.

Patient #2 is a 53-year-old Caucasian male with a history of diabetes from the maternal family. He was taking Victoza and insulin to manage his condition. His primary goal was weight loss and improving general health. He tried IF for 5 months prior to the program for 1 month with mild success, but he was not sure if he was doing it correctly. He was very motivated to make changes and decided to choose a low carbohydrate, ketogenic diet along with IF. In 4 months, he was taken off Victoza and insulin, had his A1C drop significantly from 12 to 6.5 as shown in Figure 1, and lost 7.3 pounds from 262.1 to 254.8 pounds as shown in Figure 2. Figure 6 shows the lab values of patients from the start to the end of the program. Of note, his glucose improved from 297 to 118, HDL improved from 39 to 43, and triglycerides decreased from 259 to 109. Breath ketones were positive throughout the intervention. During this program, his AGP report showed a marked improvement of his average glucose levels in Figures 7, 8.

### Patient #3

**Figure 9:**
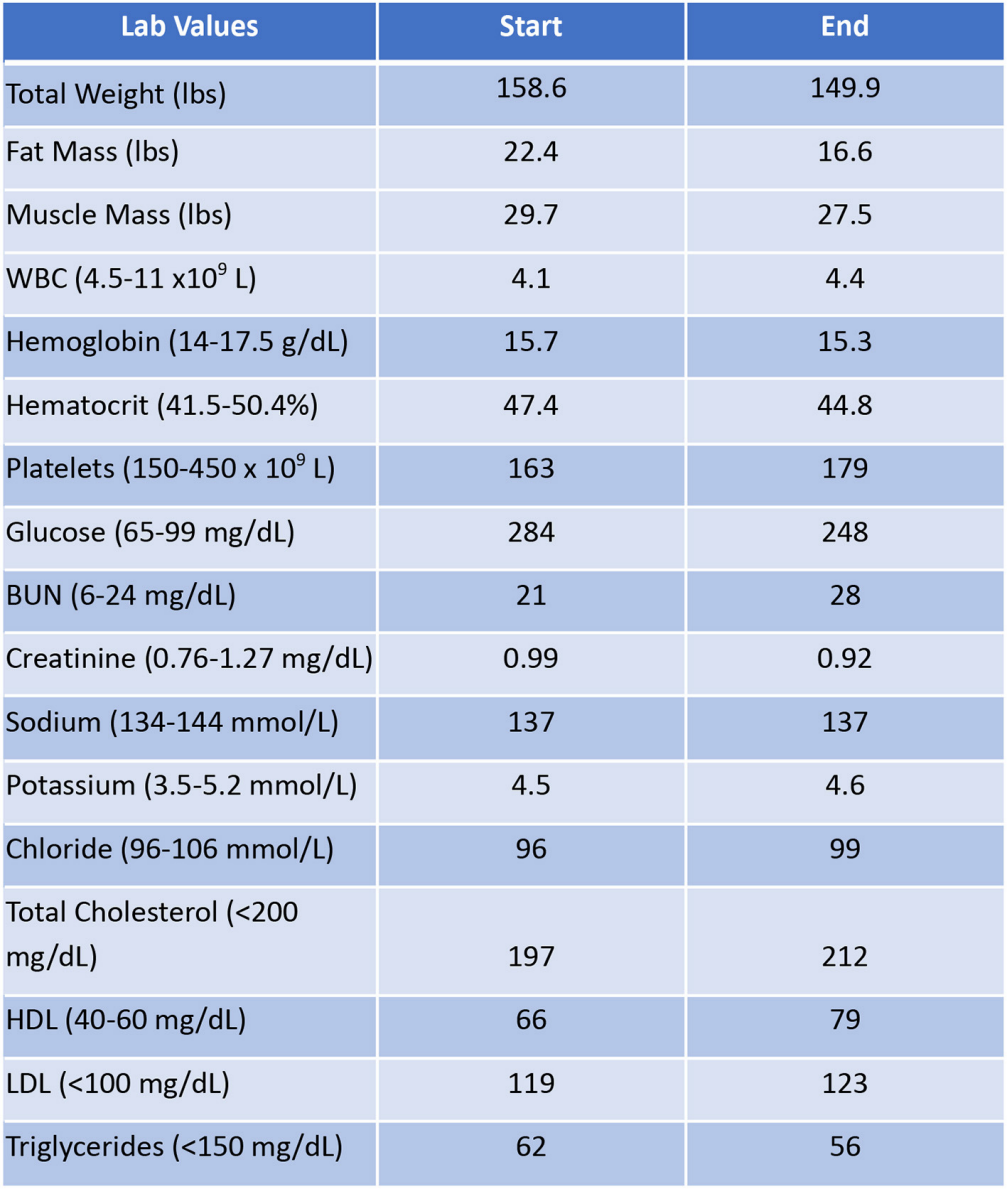
Lab values of Patient #3 at the start and the end of the program.

**Figure 10:**
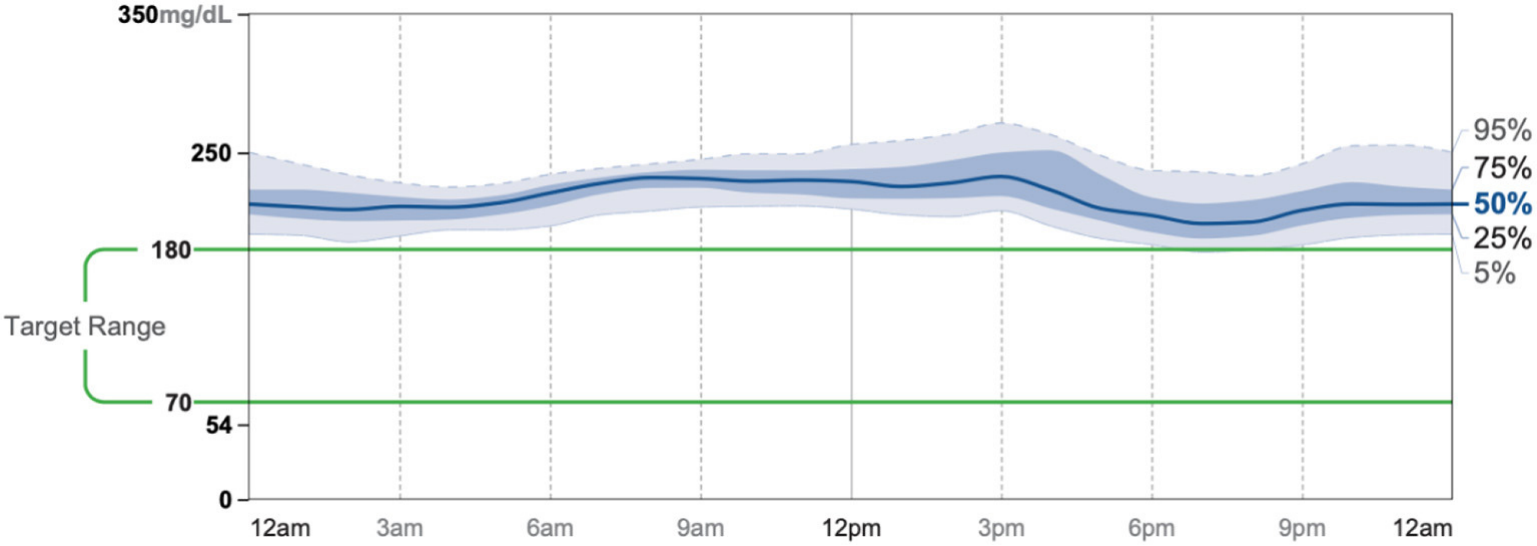
AGP 7/23-8/5.

**Figure 11:**
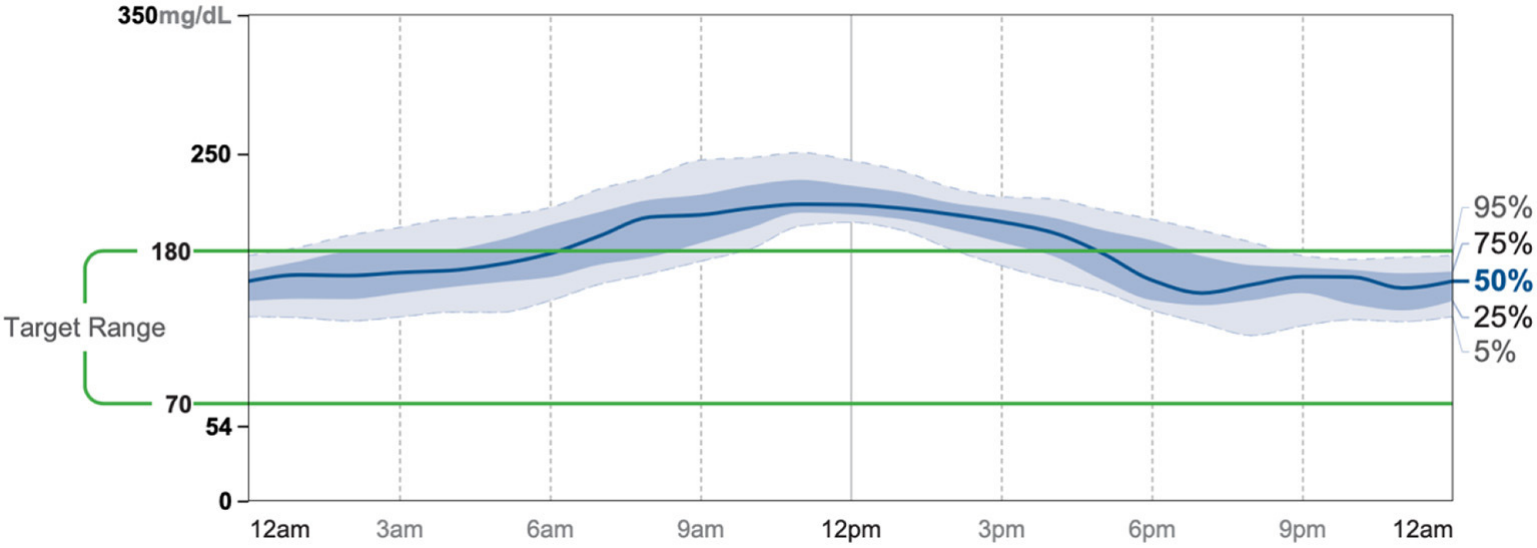
AGP 11/25-12/8.

Patient #3 is a 69-year-old Caucasian male with a history of hypertension, high cholesterol, diabetes, and alcoholism along with a maternal history of diabetes. He was taking metformin of 1,000 mg and his goal in doing this program was to improve his diabetes without the use of medications. He chose a low carbohydrate, ketogenic diet along with alternate-day fasting. In about 4 months, he was taken off metformin, had his A1C drop significantly from 11.3 to 7.5 as shown in Figure 1, and lost 8.7 pounds from 158.6 to 149.9 pounds as shown in Figure 2. Figure 9 shows the lab values of the patient from the start to the end of the program. Of note, his glucose improved from 284 to 248, HDL improved from 66 to 79 and triglycerides decreased from 62 to 56. The patient had capillary ketones checked in the office and was present throughout the program. During this program, his AGP report showed an improvement of his average glucose levels in Figures 10, 11.

## Discussion

Ketogenic diets have been used in many medical conditions, including seizure disorder, binge-eating, and hypertriglyceridemia (6, 7). It has been previously demonstrated that the long-term low-carbohydrate diets have a favorable impact on glycemia and A1C reduction (8, 9). Previously, there was a single case report of a patient with the long-term A1C reduction without significant weight loss using intermittent fasting (10). The ADA has emphasized in their guidelines that macronutrients and meal-timing should not be of focus, despite some criticism (3, 11). The ADA and various other medical organizations have recommended that the focus should be on weight reduction, energy intake reduction, and the adoption of supposed healthy dietary patterns. However, it has previously been demonstrated that factors of metabolic syndrome improve merely by lowering carbohydrates without a need for weight loss or a decrease in energy intake (12).

This is the first case series, in multiple patients with severe diabetes, demonstrating profound A1C reduction with minimal weight loss, contrary to the recommendations of the ADA. In these patients, portion size and energy restriction were not addressed at all and substantial weight loss did not occur. Patients 1 and 2 did not meet the criteria of ADA for clinically significant weight loss due to an under 5% reduction in baseline weight. Although patient #3 did lose a 5.4% reduction in weight, he was not overweight or obese. Thus, using a unique, patient-centered program that advocates for the use of a ketogenic diet with intermittent fasting resulted in a significant reduction in A1C without clinically significant weight reduction. These results call into question the emphasis of ADA on weight loss, caloric reduction, and its recommendations against focusing on macronutrient composition and meal timing. Although anecdotal, these results demonstrate the importance of both meal timing and therapeutic carbohydrate reduction on the glycemia of patients with severe diabetes, an area of further focus needed in future dietary guidelines.

## Data Availability Statement

The raw data supporting the conclusions of this article will be made available by the authors, without undue reservation.

## Ethics Statement

Written informed consent was obtained from the individuals for the publication of any potentially identifiable images or data included in this article.

## Author Contributions

KG drafted the manuscript. TK reviewed and edited manuscript drafts. All authors contributed to the article and approved the submitted version.

## Conflict of Interest

TK discloses spousal ownership of Rosette's Mix, a low-carb baking mix company, and participation in the not-for-profit LowCarbMD podcast. The remaining author declares that the research was conducted in the absence of any commercial or financial relationships that could be construed as a potential conflict of interest.
